# Retraction: Gene-Expression Changes in Cerium Chloride-Induced Injury of Mouse Hippocampus

**DOI:** 10.1371/journal.pone.0297257

**Published:** 2024-01-11

**Authors:** The PLOS ONE Editors

Following the publication of this article [1], multiple concerns were raised regarding the results and text. Specifically,

- Values in Tables 2–4 described as standard error of the mean (SEM) consistently represent 5% of the mean.
- There is minor text overlap between the Discussion section of [1] and previously published articles with no shared authors [2, 3] without attribution.
- There is substantial text overlap throughout this article with other works by the same author group that were published first or were under consideration at the same time, including [4, retracted in 5, 6, 7]. Text reuse was not cited or acknowledged in [1], and similar submitted manuscripts were not declared during the peer review process as is required by PLOS policy.
- Error bars in Figures 1, 2 and 6 do not appear uniform, and it is not clear if these error bars represent SEM as described in the legends or if they were calculated as 5% of the mean as in the tables.
- The Methods section states that Student’s t-test was used to analyse the data, however given the number of groups and conditions in each experiment, this statistical test is not appropriate.
- Tables and figures contain letters described as indicating significant differences between groups, however it is not clear which groups are being compared.

The authors did not respond to queries regarding these issues and the concerns remain unresolved.

In light of the above issues, the article does not meet the journal’s reporting and policy requirements and PLOS remains concerned about the validity and reliability of the reported quantitative results. Therefore, the *PLOS ONE* Editors retract this article. We regret that the issues were not identified before the article was published.

All authors either did not respond directly to the editorial decision or could not be reached.
